# Recognition of Transfusion-Related Acute Lung Injury in a Patient With End-Stage Liver Disease and Sepsis

**DOI:** 10.1155/crcc/7448355

**Published:** 2025-05-18

**Authors:** Matthew Viggiano, Carly Sokach, Ho-Man Yeung

**Affiliations:** Department of Medicine, Temple University Lewis Katz School of Medicine, Philadelphia, Pennsylvania, USA

## Abstract

A man in his 40s with history of decompensated cirrhosis presented with acute leg pain. On presentation, he was febrile, tachycardic, and jaundiced. Laboratory findings included leukocytosis with bandemia, anemia, elevated creatinine, hyponatremia, lactic acidosis, hyperbilirubinemia, and elevated INR. His model for end-stage liver disease-sodium (MELD-Na) score was 32. Diagnostic paracentesis did not suggest spontaneous bacterial peritonitis, but blood cultures revealed *Escherichia coli.* He received intravenous fluids, broad-spectrum antibiotics, and packed RBC. He became acutely dyspneic, 1 h into the transfusion. Over the next 36 h, he developed worsening hypoxia with infiltrates on serial chest x-rays despite adequate diuresis. He required intubation for respiratory failure and his hospital course was complicated by hepatic encephalopathy. Ultimately, he was discharged on hospital Day 18 and was retrospectively diagnosed with transfusion-related acute lung injury (TRALI) Type II. This case focuses on management of TRALI in sepsis and highlights the elevated risk of transfusion-associated reactions in liver failure patients and its mortality and morbidity.

## 1. Introduction

Gastrointestinal hemorrhage, particularly from esophageal varices, is a major cause of anemia in patients with end-stage liver disease (ESLD) [1, 2]. The American Association for the Study of Liver Diseases practice guidelines recommend conservative transfusion parameters, with a threshold hemoglobin of 7 g/dL. However, there is significant debate regarding best practice for transfusion strategies in patients with significant liver disease [3]. Furthermore, up to one-third of all hospitalized patients with cirrhosis are found to have an infection [4, 5], with a 45%–60% chance in patients with gastrointestinal bleeding [6]. Separately, incidence of multidrug-resistant infection has been found to be up to 23%–29% [7]. This can result in hypotension due to progressive splanchnic and systemic vasodilation [8]. A recent study found that 6% of septic patients admitted to the intensive care unit (ICU) had cirrhosis. Additionally, 43% of these patients had septic shock compared to 23% of noncirrhotic patients. Finally, up to 65% of patients dying during hospitalization compared to 32% of noncirrhotic patients [9]. Managing patients with ESLD, sepsis and anemia can be complex. Balancing the management of these conditions can be challenging for even experienced physicians. Given the complexity of this patient population and the frequent need for transfusion, physicians should be aware of transfusion-related complications that may occur.

Transfusion-related acute lung injury (TRALI) was redefined as of 2019. TRALI Type I is defined as the acute onset of hypoxemia (Pao_2_/FiO_2_ < 300, SpO_2_ < 90% on room air), development of noncardiogenic pulmonary edema on imaging within 6 h of transfusion, and no temporal relationship to an alternative risk factor for acute respiratory distress syndrome (ARDS). TRALI Type II patients meet the above criteria but have risk factors for ARDS and experience deteriorating respiratory status judged to be due to transfusion. This is based on stable respiratory status in 12 h before transfusion [10–12]. TRALI has emerged as the highest morbidity and mortality in patients receiving blood transfusion [13]. The true etiology of TRALI is unknown. The current proposed mechanism is the “two-hit model.” Patients have an initial predisposition with “primed” neutrophils, commonly occurring in shock, sepsis, organ damage, stress, or trauma. Subsequent activation during transfusion of blood products leads to pulmonary leukostasis, endothelial damage, capillary leak, and pulmonary edema [14]. Sepsis, chronic alcohol abuse, severe liver failure, massive transfusion, and transfusion of female donor plasma have all been associated with increased risk of the development of TRALI [15, 16]. Overall, TRALI is believed to be an underdiagnosed and underreported phenomenon, particularly in critically ill patients who have multiple risk factors for ARDS [11, 17].

TRALI has an increased incidence among patients with ESLD experiencing gastrointestinal hemorrhage. A retrospective analysis of incidence of TRALI from gastrointestinal bleeds in the ICU reported 29% of patients with ESLD developed TRALI compared to 1% without ESLD and accompanying increased mortality [18]. Sepsis and other inflammatory condition also have been associated with higher risk of TRALI, indicated by elevated levels of interleukin 8 [15]. Additionally, chronic alcohol abuse, current smoker, shock before transfusion, and positive fluid balance all increased risk of TRALI [19].

Given the increased incidence of gastrointestinal bleeding with anemia, infection, and shock in liver failure patients, transfusion is frequently considered. This case reviews a patient with the above diagnoses, investigates the risk of transfusion-associated reaction, particularly TRALI, and highlights the resulting outcomes, morbidity, and mortality of these patients.

## 2. Case Presentation

A man in his early 40s with medical history of hepatitis C cirrhosis complicated by ascites, hepatic encephalopathy, and prior subarachnoid and intraparenchymal hemorrhage, presented to the emergency department with leg pain. He takes all his prescribed medications including lactulose, rifaximin, and spironolactone. He has been adherent to scheduled weekly therapeutic paracentesis. His vital signs on arrival were T 38.1°C, heart rate 117 bpm, blood pressure 126/70 mmHg, and SpO_2_ 100% on ambient air. Physical exam was notable for normal mental status at baseline without asterixis, jaundice and sclera icterus, the lungs were clear to auscultation bilaterally, abdominal distension with fluid wave and dullness to percussion, and 2+ bilateral lower extremity edema. Laboratory data revealed WBC of 8.4 K/dL with 37% band cells, chronic macrocytic anemia with a hemoglobin at 9.1 g/dL, creatinine of 1.36 mg/dL, sodium 124 mmol/L, lactate of 2.9 mmol/L, total bilirubin of 7.8 mg/dL, direct bilirubin of 4.5 mg/dL, albumin of 1.5 mg/dL, and INR of 2.4. The MELD-Na score was 32. He was given 500-mL normal saline, albumin 1.5 g/kg/day, and vancomycin and ceftriaxone for empiric therapy for spontaneous bacterial peritonitis. He was monitored overnight but became hypotensive with a blood pressure of 91/45 mmHg. Repeat laboratory testing revealed hemoglobin 6.7 g/dL, leukocytosis, with rising creatinine and lactic acid. Patient received 2-L boluses of lactated ringers and was transfused 2 units of packed RBCs in the evening of hospital Day 2. He was started on continuous octreotide infusion and midodrine. He developed shortness of breath and hypoxia requiring 4-L nasal cannula to maintain SpO_2_ above 90%, 1 h into the transfusion. Overnight, his blood pressure improved and he remained afebrile.

**Figure 1:**
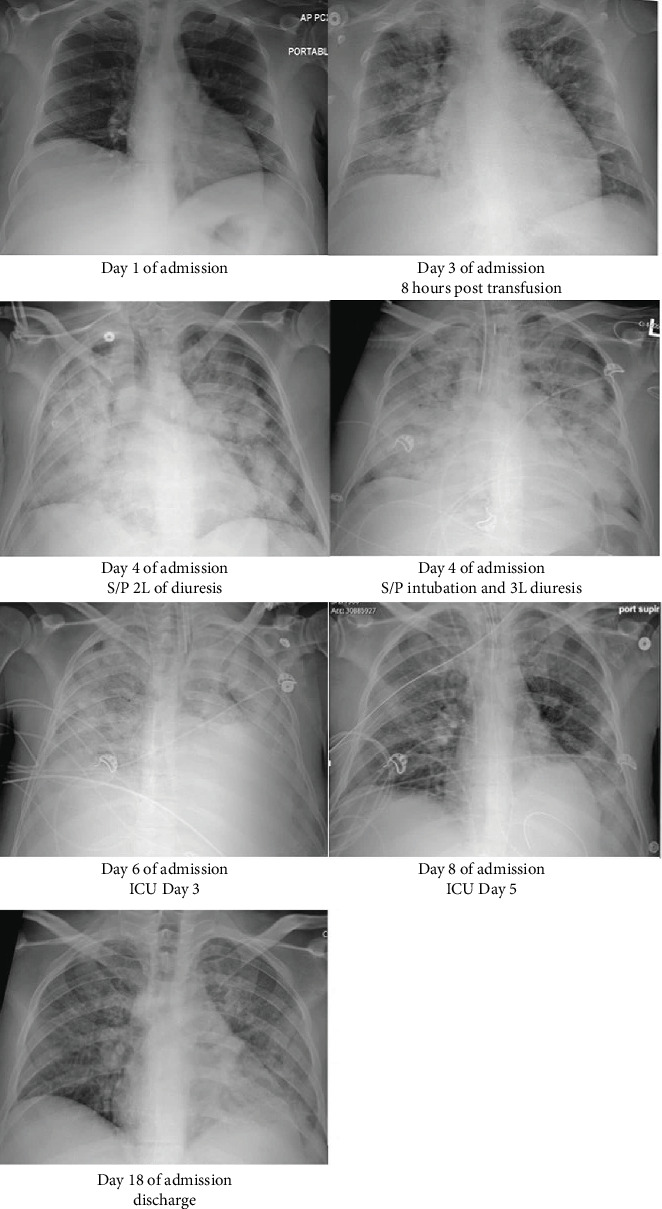
Serial chest x-rays throughout the hospital stay, showing progressive bilateral infiltrates that later improved.

On admission, urinalysis was unremarkable. EKG revealed sinus tachycardia with no significant changes from previous EKG. Initial chest x-ray showed no consolidations, infiltrates, opacities, or effusions. On hospital Day 2, he underwent an urgent paracentesis which revealed white blood cell count of 83/mm^3^ and a 75% neutrophil predominance. Findings were not consistent with spontaneous bacterial peritonitis. His blood cultures grew Gram-negative rods, later found to be *E. coli.* Transthoracic echocardiograph showed EF 50%–55%, without vegetations or pericardial effusion, and normal valvular function. The ultrasound of his abdomen revealed portal hypertension with a large volume ascites and posterior wall shadowing of the gallbladder concerning for porcelain gall bladder or cholelithiasis. Given his respiratory symptoms and hypoxia, serial chest x-rays were obtained (Figure 1).

On hospital Day 3, the patient continued to have tachypnea and hypoxia requiring high-flow nasal cannula. Chest x-ray now revealed new diffuse bilateral pulmonary infiltrates. He was given intravenous furosemide followed by intravenous bumetanide without appropriate response; thus, bumetanide infusion with metolazone was given. Noninvasive positive pressure ventilation (NIPPV) with FIO_2_ of 100% was then started for additional respiratory support.

Despite effective diuresis resulting in net urine output of 2 L, the patient continued to exhibit increased work of breathing with accessory muscle use. Arterial blood gas showed pH of 7.36 mmHg, PaO_2_ of 242 mmHg, and pCO_2_ of 44 mmol/L while on NIPPV. BNP was elevated to 1,351 pg/mL. Chest x-ray showed progressively worsened bilateral pulmonary infiltrates and opacities, despite continued aggressive diuresis. Given acute respiratory distress and likely impending respiratory failure, the patient was emergently intubated. Over the next 24 h, he continued aggressive diuresis to net output 5 L and was started on norepinephrine. Subsequent ABG showed PaO_2_/FIO_2_ (P/F) ratio of 105. Blood cultures on admission that grew *E. coli* demonstrated extended spectrum beta lactamase resistance; thus, ertapenem was started.

He remained on low tidal volume ventilation and was clinically unchanged until hospital Day 7, when his P/F ratio improved to 135 and chest x-ray began to improve. On hospital Day 13, the patient was extubated and was stable on 2-L nasal cannula. He was transferred out of the ICU but remained hospitalized for treatment of hepatic encephalopathy. He was eventually discharged to a skilled nursing facility after 16 days of hospitalization.

The patient was readmitted with encephalopathy, acute kidney injury, and hematochezia for a week and discharged to a skilled nursing facility, 3 days after discharge. He was then readmitted 3 days later after experiencing encephalopathy. During this hospitalization, the patient was transfused a unit of packed RBC, without significant events. Patient remained in hospital for 19 days. He unfortunately ultimately expired from spontaneous intracranial hemorrhage due to cirrhosis-related thrombocytopenia and coagulopathy.

## 3. Discussion

The definitive diagnosis of TRALI can be challenging, as it is predominantly based on clinical and radiographic findings. The new definitions of TRALI emphasized that the presence of ARDS risk factors does not exclude TRALI. As seen in our patient, common symptoms include inflammation, fever, and noncardiogenic pulmonary edema with pulmonary infiltrates visualized on chest x-ray [11, 12]. Hypotension has been reported but is not a consistent finding [10]. Clinicians with suspicion of TRALI should immediately discontinue transfusion of blood products. Transfusion medicine should be contacted, and laboratory evaluation of a possible transfusion reaction should be pursued.

Our case highlights the need to better understand risk factors of TRALI in ESLD patients. Evidence suggests that patients experiencing anemia from gastrointestinal bleeds with underlying ESLD have a higher risk of developing a transfusion reaction, particularly TRALI [18]. This risk compounds when patients experience sepsis or other inflammatory conditions, which frequently are seen in ESLD patients [15, 19]. Development TRALI in ESLD patients also carries an elevated risk of in hospital mortality and morbidity [18]. Our patient developed TRALI from a packed RBC transfusion. However, coagulopathy and resulting plasma or platelet containing transfusions must also be considered in ESLD patients, as they occur at an even higher rate than packed RBC transfusions [13]. Other comorbidities frequently found in liver failure patients must be considered in the clinical decision to transfuse as they increase the risk of TRALI. The decision to transfuse should be on a case-by-case basis, considering the patient's clinical status, with the risk and benefit of transfusion.

Differentiation of TRALI, TACO (transfusion-associated circulatory overload), transfusion-associated dyspnea, and sepsis is critical, particularly in patients with ESLD, due to increased risk of developing cirrhotic cardiomyopathy, valvular diseases, and left heart failure [20, 21]. This can make both diagnosis and management of hemodynamic status challenging. Sepsis management commonly requires fluid resuscitation with vasoactive support. TRALI can require fluid resuscitation or diuretic support depending on hemodynamic status. TACO treatment strategies focus on diuretic therapy to decrease preload and afterload. In the presented case, balancing sepsis with TRALI involved a level of risk, as diuretic support can improve overall oxygenation, while simultaneously worsening hypotension.

Due to the patient's worsening respiratory failure, sepsis, and anemia, a broad differential diagnosis was considered. First, given the temporal relationship to blood transfusion, we will discuss TRALI versus TACO. A case can be classified as TRALI (Type I or II), ARDS, TACO, TRALI/TACO cannot be distinguished, or transfusion-associated dyspnea [12]. Both TRALI and TACO have symptomatic overlap and can be difficult to clinically discern. Additionally, both entities are mechanistically different thus can even coexist [20]. Even with the frequent modifications to their definitions, TRALI and TACO are both still underreported and underrecognized [21] Additionally, there are no reliable biomarkers or laboratory testing that can be diagnostic for either entity [22]. Thus, recent definitions in both TRALI and TACO emphasize preexisting risk factors, symptom onset, clinical findings, and clinical improvement. This patient was found to acute severe hypoxemia by P/F ratio, bilateral pulmonary edema, within 6 h of transfusion, with sepsis as a risk factor for ARDS, which is consistent with TRALI Type II, as recently defined in the 2019 revised Delphi panel definition [12]. Furthermore, the patient's presentation did not fully meet diagnostic guidelines for TACO according to the International Society of Blood Transfusion (ISBT/IHN/AABB) 2018 definition [23]. The IHN/ISBT TACO guidelines require acute or worsening respiratory compromise and/or evidence of pulmonary edema, and three or more of the following criteria: Clinical physical exam findings and/or radiographic chest imaging or echocardiogram demonstrating worsening pulmonary edema; cardiovascular system changes include tachycardia, hypertension, widened pulse pressure, jugular venous distension, peripheral edema; and evidence of fluid overload with rapid response to diuretic therapy. Supportive biomarkers such as B-type natriuretic peptide (BNP) 1.5 times above pretransfusion value may be correlated with TACO patients. However, elevated natriuretic peptides is not considered diagnostic as it is also significantly increased in critically ill TRALI patients [22]. If the criteria for neither TRALI or TACO can be fulfilled, there exists a classification of “TRALI/TACO cannot be distinguished,” for patients in whom TRALI cannot be distinguished from TACO or in whom both conditions coexist. In our patient, he fulfilled the criteria for TRALI Type II. The preserved left heart systolic function, the lack of hypertension, lack of pleural fluid, and the lack of improvement with adequate diuresis are not suggestive of TACO. Furthermore, our patient lacked volume-sensitive risk factors typically associated with TACO including heart failure, renal failure, and advanced age.

Management of these patients focuses on supportive therapy, with oxygen supplementation being the mainstay treatment. Noninvasive respiratory support can be utilized in less severe presentations of TRALI. However, endotracheal intubation with invasive mechanical ventilation is commonly required in up to 70%–80% of cases [8, 10, 24]. Anti-inflammatory therapy such as corticosteroids is currently not recommended for treatment of TRALI [17, 25]. Patients who recover from TRALI do not have an elevated risk for recurrent episodes of transfusion reaction [17]. However, blood and plasma products should not be procured from the same donor. Transfusion of blood should not be withheld in these patients. Male donors have been found to have a lower risk of transfusion reaction [15].

The management of ESLD patients can be tenuous due to the chronic inflammatory state, risk of infection, and tenuous hemodynamic status. In situations necessitating transfusion, all the above morbidities should be considered at elevated risk of TRALI. In these patients, there are high mortality rates and poor outcomes, demonstrated by the outcome of this patient. Clinicians should be aware of the risks and maintain high suspicion for transfusion reaction, particularly with plasma and platelet containing products.

## 4. Conclusion

TRALI is often clinically diagnosed retrospectively and management can be challenging. Recognition of this entity and prompt airway management is the key to the care of TRALI. The definition and classification of TRALI was recently changed to Type I and Type II according to the 2019 Delphi panel consensus definition. “Possible TRALI” has been replaced. TACO and TRALI are clinically difficult to distinguish and may even coexist. For those patients with indistinguishable or coexisting diagnoses, there is a separate entity called “TRALI/TACO cannot be distinguished.” Liver failure patients carry an elevated risk of developing TRALI from transfusion. Liver failure patients who develop TRALI have an elevated risk of mortality and morbidity. Sepsis and chronic inflammation also increase the risk in the development of TRALI. However, the treatment of TRALI in the setting of sepsis may require conflicting treatment strategies.

## Data Availability

Data sharing is not applicable to this article as no new data were created or analyzed in this study.

## References

[1] McHutchison J. G., Manns M. P., Longo D. L. (2006). Definition and Management of Anemia in Patients Infected With Hepatitis C Virus. *Liver International*.

[2] Caldwell S. H., Hoffman M., Lisman T. (2006). Coagulation Disorders and Hemostasis in Liver Disease. *Hepatology*.

[3] Garcia-Tsao G., Sanyal A. J., Grace N. D., Carey W. D., the Practice Guidelines Committee of the American Association for the Study of Liver Diseases and the Practice Parameters Committee of the American College of Gastroenterology (2007). Prevention and Management of Gastroesophageal Varices and Variceal Hemorrhage in Cirrhosis. *The American Journal of Gastroenterology*.

[4] Borzio M., Salerno F., Piantoni L. (2001). Bacterial Infection in Patients With Advanced Cirrhosis: A Multicentre Prospective Study. *Digestive and Liver Disease*.

[5] Bunchorntavakul C., Chamroonkul N., Chavalitdhamrong D. (2016). Bacterial Infections in Cirrhosis: A Critical Review and Practical Guidance. *World Journal of Hepatology*.

[6] Thalheimer U., Triantos C. K., Samonakis D. N., Patch D., Burroughs A. K. (2005). Infection, Coagulation, and Variceal Bleeding in Cirrhosis. *Gut*.

[7] Fernández J., Prado V., Trebicka J. (2019). Multidrug-Resistant Bacterial Infections in Patients With Decompensated Cirrhosis and With Acute-on-Chronic Liver Failure in Europe. *Journal of Hepatology*.

[8] Iwakiri Y., Groszmann R. J. (2006). The Hyperdynamic Circulation of Chronic Liver Diseases: From the Patient to the Molecule. *Hepatology*.

[9] Chebl R. B., Tamim H., Sadat M., Qahtani S., Dabbagh T., Arabi Y. M. (2021). Outcomes of Septic Cirrhosis Patients Admitted to the Intensive Care Unit. *Medicine (Baltimore)*.

[10] Popovsky M. A., Moore S. B. (1985). Diagnostic and Pathogenetic Considerations in Transfusion-Related Acute Lung Injury. *Transfusion*.

[11] Kleinman S., Caulfield T., Chan P. (2004). Toward an Understanding of Transfusion-Related Acute Lung Injury: Statement of a Consensus Panel. *Transfusion*.

[12] Vlaar A. P. J., Toy P., Fung M. (2019). A Consensus Redefinition of Transfusion-Related Acute Lung Injury. *Transfusion*.

[13] US Food and Drug Adminsitration (2008). *Fatalities Reported to FDA Following Blood Collection and Transfusion: Fiscal Report 2008*.

[14] Otrock Z. K., Liu C., Grossman B. J. (2017). Transfusion-Related Acute Lung Injury Risk Mitigation: An Update. *Vox Sanguinis*.

[15] Toy P., Gajic O., Bacchetti P. (2012). Transfusion-Related Acute Lung Injury: Incidence and Risk Factors. *Blood*.

[16] Roubinian N. (2018). TACO and TRALI: Biology, Risk Factors, and Prevention Strategies. *Hematology Am Soc Hematol Educ Program*.

[17] Looney M. R., Gropper M. A., Matthay M. A. (2004). Transfusion-Related Acute Lung Injury. *Chest*.

[18] Benson A. B., Austin G. L., Berg M. (2010). Transfusion-Related Acute Lung Injury in ICU Patients Admitted With Gastrointestinal Bleeding. *Intensive Care Medicine*.

[19] Toy P., Bacchetti P., Grimes B. (2015). Recipient Clinical Risk Factors Predominate in Possible Transfusion-Related Acute Lung Injury. *Transfusion*.

[20] Gajic O., Gropper M. A., Hubmayr R. D. (2006). Pulmonary Edema After Transfusion: How to Differentiate Transfusion-Associated Circulatory Overload From Transfusion-Related Acute Lung Injury. *Critical Care Medicine*.

[21] Van den Akker T. A., Grimes Z. M., Friedman M. T. (2021). Transfusion-Associated Circulatory Overload and Transfusion-Related Acute Lung Injury. *American Journal of Clinical Pathology*.

[22] Semple J. W., Rebetz J., Kapur R. (2019). Transfusion-Associated Circulatory Overload and Transfusion-Related Acute Lung Injury. *Blood*.

[23] Wiersum-Osselton J. C., Whitaker B., Grey S. (2019). Revised International Surveillance Case Definition of Transfusion-Associated Circulatory Overload: A Classification Agreement Validation Study. *The Lancet Haematology*.

[24] Vlaar A. P., Binnekade J. M., Prins D. (2010). Risk Factors and Outcome of Transfusion-Related Acute Lung Injury in the Critically Ill: A Nested Case-Control Study^∗^. *Critical Care Medicine*.

[25] Steinberg K. P., Hudson L. D., Goodman R. B. (2006). Efficacy and Safety of Corticosteroids for Persistent Acute Respiratory Distress Syndrome. *The New England Journal of Medicine*.

